# Combined influence of depressive symptoms and systemic inflammation on all-cause and cardiovascular mortality: evidence for differential effects by gender in the English Longitudinal Study of Ageing

**DOI:** 10.1017/S003329171800209X

**Published:** 2018-09-17

**Authors:** Samantha Lawes, Panayotes Demakakos, Andrew Steptoe, Glyn Lewis, Livia A. Carvalho

**Affiliations:** 1 Department of Epidemiology and Public Health, University College London, London, UK; 2 Division of Psychiatry, University College London, London, UK; 3 Department of Clinical Pharmacology, William Harvey Research Institute, Queen Mary University of London, London, UK

**Keywords:** Cardiovascular disease, depressive symptoms, inflammation, mortality

## Abstract

**Background:**

Depressive symptoms and inflammation are risk factors for cardiovascular disease (CVD) and mortality. We investigated the combined association of these factors with the prediction of CVD and all-cause mortality in a representative cohort of older men and women.

**Methods:**

We measured C-reactive protein (CRP) and depressive symptoms in 5328 men and women aged 52–89 years in the English Longitudinal Study of Ageing. Depressive symptoms were measured using the eight-item Centre for Epidemiological Studies Depression Scale. CRP was analysed from peripheral blood. Mortality was ascertained from national registers and associations with depressive symptoms and inflammation were estimated using Cox proportional hazard models.

**Results:**

We identified 112 CVD related deaths out of 420 all-cause deaths in men and 109 CVD related deaths out of 334 all-cause deaths in women over a mean follow-up of 7.7 years. Men with both depressive symptoms and high CRP (3–20 mg/L) had an increased risk of CVD mortality (hazard ratio; 95% confidence interval: 3.89; 2.04–7.44) and all-cause mortality (2.40; 1.65–3.48) after adjusting for age, socioeconomic variables and health behaviours. This considerably exceeds the risks associated with high CRP alone (CVD 2.43; 1.59–3.71, all-cause 1.49; 1.20–1.84). There was no significant increase in mortality risk associated with depressive symptoms alone in men. In women, neither depressive symptoms or inflammation alone or the combination of both significantly predicted CVD or all-cause mortality.

**Conclusions:**

The combination of depressive symptoms and increased inflammation confers a considerable increase in CVD mortality risk for men. These effects appear to be independent, suggesting an additive role.

## Introduction

A number of population-based studies have reported that people with depressive symptoms are at greater risk of mortality (Wulsin *et al*., 1999; Schulz *et al*., 2002; Wulsin *et al*., 2005; Lasserre *et al*., 2016), particularly from cardiovascular disease (CVD) (Laursen *et al*., 2007; Wu and Kling, 2016; Correll *et al*., 2017). Even patients with sub-clinical levels of depressive symptoms, face increased mortality risk (Russ *et al*., 2012). A robust literature demonstrates that depressive symptoms increase CVD risk in initially healthy people (Cuijpers and Smit, 2002; Rugulies, 2002; Wulsin and Singal, 2003; Nicholson *et al*., 2006; Van der Kooy *et al*., 2007; Gan *et al*., 2014) and CVD mortality in those with established disease (Barth *et al*., 2004; van Melle *et al*., 2004; Nicholson *et al*., 2006; Meijer *et al*., 2011; Meijer *et al*., 2013). Many studies have specifically reported an association between depressive symptoms and mortality in older people. Incident (Penninx *et al*., 1998; Vinkers *et al*., 2004; Teng *et al*., 2013), intermittent (Geerlings *et al*., 2002) and chronic depression (Geerlings *et al*., 2002; Schoevers *et al*., 2009; Teng *et al*., 2013) increase all-cause and CVD mortality in this age group. However, these findings are not entirely consistent, with some studies reporting no association (Callahan *et al*., 1998; Cuijpers, 2001; Hybels *et al*., 2002; McCusker *et al*., 2006). Both persistence and severity of depressive symptoms are associated with increased mortality risk in older people (White *et al*., 2015, 2016).

Extensive evidence also exists for an association between depressive symptoms and inflammation (Raison *et al*., 2006; Irwin and Miller, 2007; Miller *et al*., 2009; Miller and Raison, 2016). Longitudinal studies have reported an association between depressive symptoms and inflammatory biomarkers in otherwise healthy people (Gimeno *et al*., 2009; Hamer *et al*., 2009; Copeland *et al*., 2012; Au *et al*., 2015; Zalli *et al*., 2015; Bell *et al*., 2017). In addition, several meta-analyses show higher levels of inflammation in depressed patients compared with healthy controls (Howren *et al*., 2009; Dowlati *et al*., 2010; Liu *et al*., 2012; Valkanova *et al*., 2013; Eyre *et al*., 2016). A recent cumulative meta-analysis confirmed the robust nature of the depression–inflammation relationship, showing evidence of increased circulating interleukin-6 and C-reactive protein (CRP) in depressed compared with non-depressed individuals (Haapakoski *et al*., 2015). In patients with CVD, the prevalence of depression is four times higher than in people from the general population (Thombs *et al*., 2006). Depression also shows a high co-morbidity with a number of major chronic inflammatory or autoimmune disorders, such as rheumatoid arthritis, multiple sclerosis and diabetes type 1 and 2 (Maes *et al*., 2012). However, it is unclear whether the increased mortality risk in depressed people is due to inflammation.

In light of the well-established role of inflammatory processes in both the pathogenesis of atherosclerosis and the prediction of cardiac events (Shimbo *et al*., 2005; Golia *et al*., 2014; Biasucci *et al*., 2017), it has been proposed that inflammation may be mediating the association between depression and mortality, particularly in relation to CVD. However, evidence linking inflammation and depressive symptoms with the development of CVD is inconsistent. A population-based study reported that depressive symptoms and CRP interact in the prediction of coronary heart disease (CHD) in men (Ladwig *et al*., 2005). However, other studies have demonstrated that depressive symptoms increase CVD risk independently of inflammatory biomarkers (Empana *et al*., 2005; Nabi *et al*., 2008; Surtees *et al*., 2008; Davidson *et al*., 2009; Hamer *et al*., 2011), whilst several studies have reported minimal effects of mediation (Vaccarino *et al*., 2007; Kop *et al*., 2010; Hiles *et al*., 2015; Hughes *et al*., 2016). To our knowledge, only one study has investigated the combined effect of depressive symptoms and elevated inflammation on CVD development. Ladwig *et al*. (2005) showed that combined depressed mood and high CRP (>3 mg/L) conferred a significantly greater risk of future cardiac events than either depressed mood or high CRP alone in older men.

Gender differences have also been observed in the association between late-life depression and mortality. Several studies report a greater mortality risk in men (Anstey and Luszcz, 2002; Ryan *et al*., 2008; Jeong *et al*., 2013; Diniz *et al*., 2014), particularly for incident depression (Teng *et al*., 2013) and CVD (Penninx *et al*., 1998; Wulsin *et al*., 1999); whilst women appear to have increased mortality risk in the presence of chronic or severe depressive symptoms (Ryan *et al*., 2008; Teng *et al*., 2013). An emerging literature also suggests women are more likely to present with inflammation when depressed (Bell *et al*., 2017), potentially due to higher reactivity to stressful stimuli (Piccinelli and Wilkinson, 2000). Whether the inflammation and depression link is particularly important for women is still unknown.

In this study, we used data from the English Longitudinal Study of Ageing (ELSA) to investigate the combined effects of depressive symptoms and inflammation on CVD and all-cause mortality risk in older men and women. In particular, we sought to investigate whether depressive symptoms moderate the mortality risk associated with increased inflammation or whether inflammation could be mediating the association between depressive symptoms and mortality. In light of previous findings, we hypothesise that people with both depressive symptoms and inflammation will have significantly greater mortality risk than people with depressive symptoms or inflammation alone.

## Materials and methods

### Study population

The ELSA is a prospective study of a representative sample of community-dwelling people aged 50 and over living in England. It collects health, social and economic data. The study commenced in 2002, and the sample has been followed up every 2 years. Data are collected using computer-assisted personal interviews and self-completion questionnaires, with additional nurse visits for the assessment of biomarkers every 4 years. Wave 1 included a baseline interview and took place in 2002–2003. Wave 2 took place in 2004–2005 and consisted of an interview and a health examination that included a collection of blood samples. At wave 1, the sample consisted of 11 391 study members and was deemed to be nationally representative. For more information on ELSA see http://www.elsa-project.ac.uk/.

Our study included 5328 people, aged 52–89 years, from an initial cohort of 8670 who participated in the interview at wave 2. We excluded 1084 individuals who did not participate in the health examination survey, 134 who did not consent for their vital status data to be included, 1411 who were unable to provide a blood sample and 330 individuals whose CRP values were unavailable or not reliable. The latter included samples which were lost in the post, received later than 5 days after collection, considered unusable by the laboratory or of insufficient amount to be analysed. Detailed information about how the ELSA data were collected and processed into their current format and about how each variable was coded is available at https://discover.ukdataservice.ac.uk/series/?sn=200011. We also excluded 155 participants from the analysis because their CRP levels were ⩾20 ml/L, allowing for the elimination of individuals with acute inflammation, while another 229 participants were excluded because of missing values in the covariates.

Participants included and excluded from the analysis did not differ significantly in terms of sex. The group included in the analysis was younger, less likely to be depressed, more likely to be married, more educated and wealthier than the group who was excluded.

### Assessment of inflammation

Blood samples were taken by the study nurse at wave 2 and serum CRP was analysed by Royal Victoria Infirmary, Newcastle. High sensitivity plasma CRP level was dichotomised into two categories: <3 mg/L was defined as normal and 3–20 mg/L was defined as high. This cut off point is based on guidelines from the Centre for Disease Control and Prevention and the American Heart Association, suggesting that plasma CRP values of >3.0 mg/L might be predictive of CVD (Pearson *et al*., 2003).

### Assessment of depressive symptoms

Depressive symptoms were measured at wave 2 (2004–2005) using the eight-item Centre for Epidemiological Studies Depression Scale 8 (CES-D8) which is a self-report questionnaire designed to measure depressive symptomatology in the general population (Radloff, 1977). Respondents were asked how often they felt depressed, felt that everything was an effort, slept restlessly, were happy, felt lonely, enjoyed life, felt sad, and could not get going. The two positive items (‘was happy’ and ‘enjoyed life’) are reverse coded, so a higher score here also indicates a more depressed mood. We subsequently derived a summary CES-D score by adding responses to all eight dichotomous questions (possible range: 0–8). The exact wording of the different items can be found in appendix 1. The eight-item abbreviated version of the CES-D has been widely used, is internally consistent, has been validated in both the general population (Van de Velde *et al*., 2009) and older adults, and shows a comparable construct of depression across 11 different countries (Missinne *et al*., 2014). The presence of depressive symptoms was defined as CES-D ⩾4 as per previous publications (Steffick, 2000; Hamer *et al*., 2012; Mhaoláin *et al*., 2012; Demakakos *et al*., 2013; Malgaroli *et al*., 2017). This conservative threshold has been found to produce comparable results to the >16 cut off on the well-validated 20-item CES-D scale (Radloff, 1977; Steffick, 2000).

### Depressive symptoms and inflammation as a combined variable

In order to ascertain whether the combination of depressive symptoms and inflammation predicts mortality, the variables based on wave 2 assessments were combined and four new variables were computed: ‘no depressive symptoms/low inflammation’, ‘no depressive symptoms/high inflammation’, ‘depressive symptoms/low inflammation’ and ‘depressive symptoms/high inflammation’.

## Mortality

Mortality was ascertained for a mean 7.7 year period for consenting study members (5328) by linking to the UK National Health Service mortality register up until 12 November 2015. In England, all deaths need to be registered within 5 days, therefore participants not registered as dead were assumed to be alive. Deaths were classified according to International Classification of Diseases (ICD) 10th Edition. Deaths with ICD10 codes I00 to I99 were classified as cardiovascular deaths.

## Covariates

All covariates were collected at wave 2 (2004–2005) with the exception of education and sex which were collected at wave 1. All covariates where determined by self-report, with the exception of body mass index (BMI). Age was treated as a continuous variable. Socioeconomic status (SES) was operationalised by using marital status (married/cohabiting *v.* not married/single/divorced), level of education (degree/higher/A-level, GCSE/O-level/other, no qualifications) and total wealth in tertiles. Total wealth was defined from the sum of financial, physical (e.g. businesses, land) and housing wealth, minus debts and pension payments. Health behaviours included: smoking (never smoked, ex-smoker, current smoker) and BMI (<25 kg/m^2^, 25–29.99 kg/m^2^, >30 + kg/m^2^) (Banks *et al*., 2006). The presence of chronic diseases were added as separate variables and defined as yes/no. They were calculated as lifetime self-reported physician diagnoses of chronic conditions (i.e. CVD (myocardial infarction and stroke), chronic lung disease, cancers (of any site) and emotional, nervous and psychiatric problems).

The covariates have been included because they have all been shown to be associated with mortality and are therefore potential confounders (Marmot *et al*., 2012; Wong, 2014; Pletcher and Moran, 2017). Furthermore, health behaviours such as smoking and BMI were included as they have been shown to mediate the relationship between depression and mortality (Joynt *et al*., 2003).

### Statistical analysis

Differences between the characteristics of the participants included and excluded from the analysis were analysed. Baseline characteristics were analysed by depressive symptoms and inflammation levels. The association of depressive symptoms and inflammation levels (separately and in combination) with CVD and all-cause mortality were assessed by Cox proportional hazards regression models. The proportionality assumption was tested using Nelson–Aalen cumulative hazard curves and Schoenfeld residuals. We inspected the plots and the global test (*p* = 0.8052) confirming that we do not have a violation of the proportional assumption. Survival time was measured in months, from the date of interview in wave 2 (2004–2005) to the date of death or 12 November 2015, whichever was first. Kaplan Meier survival curves are available online (online Supplementary Fig. S1).

We investigated whether there were significant interactions between age or sex and depression/inflammation categories in order to determine whether the association between depression/inflammation levels and mortality varied according to age or between men and women. We used the likelihood ratio test to compare the goodness of fit of a stratified model. We found no interaction between sex and depressive symptoms, however, we did find that the association between inflammation and mortality varied significantly by sex (*p* value = 0.013). There was also an age interaction between inflammation and mortality, however, this disappeared once we stratified our analysis by sex, therefore, we only present the sex-stratified analyses. We first fitted a basic unadjusted model, which was followed by an age-adjusted model. We then additionally adjusted for socioeconomic variables, health behaviours and chronic diseases.

To investigate moderation we tested whether a multiplicative interaction between depressive symptoms and inflammation was significantly associated with mortality. To investigate mediation, we first examined the association between depressive symptoms and mortality and then added inflammation to see how much of the association was explained. All analyses were performed using STATA 13.0 (StataCorp LP, College Station Texas).

## Results

There were 420 all-cause male deaths (including 112 CVD related) over mean of 7.6 years follow-up and 334 all-cause female deaths (including 109 CVD related) over a mean of 7.8 years follow-up. Out of a total of 5328 people, we identified 420 all-cause deaths in men and 334 in women during 18 594 and 22 519 person-years, respectively.

Table 1 represents social-demographic characteristics in depression/inflammation categories in men and women separately. Men and women with concurrent depressive symptoms and high inflammation were more likely to be poorer, less educated, more likely to smoke, have a higher BMI and were more likely to have chronic lung disease and emotional, nervous and psychiatric problems. There were no significant overall differences in the frequency of CVD or cancer between depression/inflammation levels in either men or women. (Table 1).

**Table 1.** Baseline characteristics of men and women aged 52–89 years by depressive symptoms and inflammation level

	Men					Women				
	Depressive symptoms/inflammation level									
	No depressive symptoms/ normal CRP	No depressive symptoms/high CRP	Depressive symptoms/normal CRP	Depressive symptoms/high CRP	*p* value	No depressive symptoms/normal CRP	No depressive symptoms/high CRP	Depressive symptoms/normal CRP	Depressive symptoms/high CRP	*p* value
Number	1551	668	132	98		1542	864	275	198	
Mean age (SD) (years)	64.9 (8.8)	67 (9.1)	64.5 (9.1)	66.9 (9.9)	<0.001	65 (9.0)	66.5 (9.2)	67.3 (10.3)	67.4 (9.7)	<0.001
Marital status (%)										
Married	1252 (80.7)	501 (75)	79 (59.9)	60 (61)	<0.001	1023 (66.3)	530 (61.3)	128 (46.6)	83 (41.9)	<0.001
Not married	299 (19.3)	167 (25)	53 (40)	38 (39)		519 (33.7)	334 (38.7)	147 (53.5)	115 (58.1)	
Education (%)										
Degree/Higher/A-level	700 (45.1)	228 (34.1)	43 (32.6)	27 (27.6)	<0.001	458 (29.7)	191 (22.1)	51 (18.6)	39 (19.7)	<0.001
GCSE/O-level/Other qualification	444 (28.6)	203 (30.4)	37 (28)	27 (27.6)		555 (36)	281 (32.5)	98 (35.6)	53 (26.8)	
No qualification	407 (26.2)	237 (35.5)	52 (39.4)	44 (44.9)		529 (34.3)	392 (45.4)	126 (45.8)	106 (53.54)	
Total wealth (%)										
Richest	688 (44.4)	203 (30.4)	40 (30.3)	17 (17.4)	<0.001	681 (44.2)	228 (26.4)	73 (26.6)	28 (14.1)	<0.001
Intermediate	531 (34.2)	252 (37.7)	35 (26.5)	35 (35.7)		486 (31.5)	323 (37.4)	89 (32.4)	82 (41.4)	
Poorest	332 (21.4)	213 (31.9)	57 (43.2)	46 (46.9)		375 (24.3)	313 (36.2)	113 (41.1)	88 (44.4)	
Smoking status (%)										
Never a smoker	496 (32)	149 (22.3)	34 (25.8)	15 (15.3)	<0.001	743 (48.2)	379 (43.9)	107 (38.9)	65 (32.8)	<0.001
Ex-smoker	894 (57.6)	383 (57.3)	73 (55.3)	57 (58.2)		634 (41.1)	355 (41.1)	126 (45.8)	79 (39.9)	
Current smoker	161 (10.4)	136 (20.4)	25 (18.9)	26 (26.5)		165 (10.7)	130 (15.1)	42 (15.3)	54 (27.3)	
Body Mass Index										
<25 kg/m^2^ (%)	403 (26.8)	123 (19.3)	35 (27.8)	20 (23.5)	<0.001	606 (40.5)	121 (14.9)	98 (37)	32 (17.5)	<0.001
25–29.99 kg/m^2^ (%)	777 (51.7)	306 (48.1)	59 (46.8)	32 (37.7)		628 (42)	285 (35)	113 (42.6)	65 (35.5)	
>30 + kg/m^2^ (%)	323 (21.5)	207 (32.6)	32 (25.4)	33 (38.8)		262 (17.5)	408 (50.1)	54 (20.4)	86 (47)	
Chronic disease										
CVD (% yes)	115 (7.41)	61 (9.13)	14 (10.61)	14 (14.29)	0.052	48 (3.11)	31 (3.59)	14 (5.09)	14 (7.07)	0.026
Cancer (% yes)	85 (5.48)	46 (6.89)	7 (5.30)	11 (11.22)	0.093	139 (9.01)	58 (6.73)	26 (9.49)	14 (7.07)	0.191
Lung disease^a^ (% yes)	78 (5.03)	70 (10.48)	10 (7.58)	18 (18.37)	<0.001	55 (3.57)	69 (8.00)	31 (11.31)	33 (16.67)	<0.001
Psychiatric^b^ (% yes)	81 (5.22)	32 (4.79)	25 (18.94)	22 (22.45)	<0.001	159 (10.31)	81 (9.40)	58 (21.17)	51 (25.76)	<0.001

SD = standard deviation. Statistical tests examined the associations of demographic variables with depressive symptoms/inflammation levels. ANOVA tests were used for continuous variables and Chi-square tests were used for categorical variables.

a Chronic lung disease.

b Emotional, nervous or psychiatric diseases.

### Depressive symptoms and inflammation as a combined predictor of CVD mortality

**Fig. 1.**
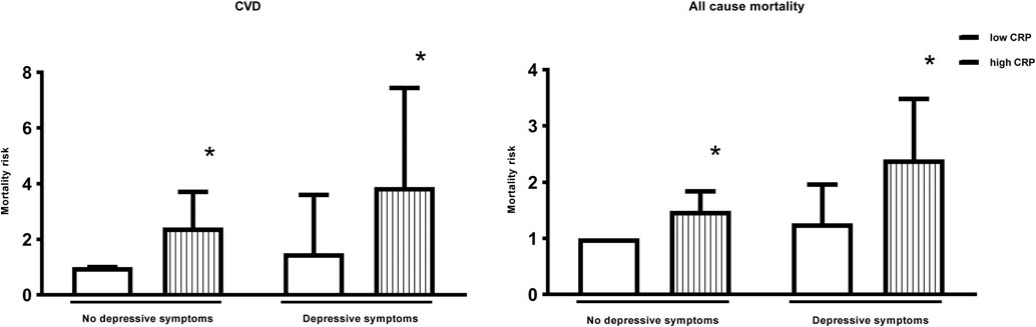
Adjusted hazard ratios for CVD and all-cause mortality according to levels of depressive symptoms and inflammation in men. * Statistically significant at *P*<0.001.CVD = Cardiovascular disease; CRP = C-reactive protein. Hazard ratios are adjusted for age, socioeconomic variables (marital status, level of education, total wealth), health behaviours (smoking, body mass index) and chronic diseases (cardiovascular disease, cancers, chronic lung disease and emotional, nervous and psychiatric problems). n = 5,328.

In men, depressive symptoms alone were not associated with any significant increase in the risk of death, whilst high inflammation was associated with a 238% (HR: 3.38; 95% CI 2.23–5.10) increased risk. Men with both depressive symptoms and high inflammation had a 584% (HR: 6.84; 95% CI 3.71–12.6) increased CVD mortality risk. This association remained significant after adjustment for age, SES and health behaviours, with men who had both depressive symptoms and high inflammation demonstrating a 289% (HR: 3.89; 95% CI 2.04–7.44) increased risk of death (*p* value <0.001) (Table 2 and Fig. 1). In women, the associations were more modest and failed to reach significance (Table 2).

**Table 2.** Association between depressive symptoms/inflammation levels and all-cause and cardiovascular mortality by sex+

	Men				Women			
	Depressive symptoms/inflammation level							
	No depressive symptoms/normal CRP	No depressive symptoms/high CRP	Depressive symptoms/normal CRP	Depressive symptoms/high CRP	No depressive symptoms/normal CRP	No depressive symptoms/high CRP	Depressive symptoms/normal CRP	Depressive symptoms/high CRP
Model								
All-cause mortality								
Model 1 HR (95% CI, *p* values)^a^	1.00 (reference)	1.91 (1.55–2.36) <0.001	1.42 (0.93–2.17) 0.104	3.41 (2.39–4.86) <0.001	1.00 (reference)	1.48 (1.16–1.90) 0.002	1.85 (1.32–2.59) <0.001	1.89 (1.29–2.76) 0.001
Model 2 HR (95% CI, *p* values)^b^	1.00 (reference)	1.63 (1.32–2.00) <0.001	1.47 (0.97–2.25) 0.072	3.03 (2.12–4.32) <0.001	1.00 (reference)	1.23 (0.96–1.57) 0.104	1.34 (0.95–1.87) 0.093	1.44 (0.98–2.10) 0.060
Model 3 HR (95% CI, *p* values)^c^	1.00 (reference)	1.54 (1.25–1.90) <0.001	1.29 (0.84–1.98) 0.242	2.59 (1.80–3.74) <0.001	1.00 (reference)	1.18 (0.92–1.51) 0.199	1.27 (0.90–1.78) 0.168	1.30 (0.89–1.92) 0.176
Model 4 HR (95% CI, *p* values)^d^	1.00 (reference)	1.49 (1.20–1.84) <0.001	1.27 (0.83–1.96) 0.273	2.40 (1.65–3.48) <0.001	1.00 (reference)	1.09 (0.84–1.41) 0.536	1.18 (0.84–1.66) 0.336	1.15 (0.77–1.70) 0.501
Model 5 HR (95% CI, *p* values)^e^	1.00 (reference)	1.46 (1.18–1.81) <0.001	1.24 (0.80–1.90) 0.337	2.09 (1.43–3.07) <0.001	1.00 (reference)	1.11 (0.85–1.44) 0.436	1.12 (0.79–1.58) 0.336	1.10 (0.74–1.65) 0.629
Cardiovascular disease mortality								
Model 1 HR (95% CI, *p* values)^a^	1.00 (reference)	3.38 (2.23–5.10) <0.001	1.86 (0.79–4.38) 0.159	6.84 (3.71–12.6) <0.001	1.00 (reference)	1.45 (0.96–2.2) 0.079	1.27 (0.66–2.44) 0.417	1.29 (0.61–2.71) 0.508
Model 2 HR (95% CI, *p* values)^b^	1.00 (reference)	2.81 (1.86–4.26) <0.001	1.91 (0.81–4.51) 0.140	5.78 (3.13–10.68) <0.001	1.00 (reference)	1.14 (0.75–1.73) 0.540	0.81 (0.42–1.56) 0.531	0.93 (0.44–1.97) 0.857
Model 3 HR (95% CI, *p* values)^c^	1.00 (reference)	2.56 (1.68–3.89) <0.001	1.51 (0.63–3.62) 0.354	4.50 (2.39–8.47) <0.001	1.00 (reference)	1.09 (0.71–1.67) 0.685	0.76 (0.39–1.46) 0.407	0.84 (0.40–1.80) 0.658
Model 4 HR (95% CI, *p* values)^d^	1.00 (reference)	2.43 (1.59–3.71) <0.001	1.50 (0.63–3.60) 0.361	3.89 (2.04–7.44) <0.001	1.00 (reference)	0.92 (0.59–1.44) 0.715	0.69 (0.35–1.33) 0.267	0.69 (0.32–1.50) 0.353
Model 5 HR (95% CI, *p* values)^e^	1.00 (reference)	2.42 (1.58–3.69) <0.001	1.44 (0.60–3.46) 0.413	3.25 (1.66–6.40) 0.001	1.00 (reference)	0.96 (0.62–1.50) 0.866	0.69 (0.35–1.35) 0.275	0.68 (0.32–1.49) 0.338

HR = hazard ratio; CI = confidence interval; CRP = C-reactive protein. Cox regression survival analysis models, stratified by sex, are adjusted as follows:

a Inflammation and depressive symptoms as main effects.

b As model 1, plus adjustment for age.

c As model 2, plus adjustment for socioeconomic variables (marital status, level of education, total wealth).

d As model 3, plus adjustment for health behaviours (smoking, body mass index).

e A model 4, plus individually adjustment for chronic diseases (cardiovascular disease, cancers, chronic lung disease and emotional, nervous and psychiatric problems).

### Depressive symptoms and inflammation as a combined predictor of all-cause mortality

In men, the direction of results was similar for all-cause mortality. Depressive symptoms alone were not associated with a significant increase in the risk of death, whilst high inflammation was associated with a 91% (HR: 1.91; 95% CI 1.55–2.36) increased risk, compared with men with neither. Men with both depressive symptoms and high inflammation had a 241% (HR: 3.41; 95% CI 2.39–4.86) increased mortality risk. This association was attenuated after adjustment for age, SES and smoking. However, the association remained significant, with men who had both depressive symptoms and high inflammation demonstrating a 140% (HR: 2.40; 95% CI 1.65–3.48) increased risk of death (Fig. 1).

The results for women were different. In the unadjusted model, both depressive symptoms and high inflammation separately increased the risk of death by 85% (HR: 1.85; 95% CI 1.32–2.59) and 48% (HR: 1.48; 95% CI 1.16–1.90), respectively. The combination of both depressive symptoms and high inflammation increased the risk to 89% (HR: 1.89; 95% CI 1.29–2.76), only marginally more than depressive symptoms alone. The increased risk of depressive symptoms and high inflammation to all-cause mortality in women was explained by age as after adjustments the risk was no longer significant (Table 2).

### Effects of moderation and mediation

Moderation analysis showed that an interaction term of depressive symptoms by inflammation was not significantly associated with mortality in all categories: all-cause mortality in men (*p* = 0.426); all-cause mortality in women (*p* = 0.155); CVD mortality in men (*p* = 0.868) and CVD mortality in women (*p* = 0.481). This suggests that the mortality risk conferred by increased levels of inflammation is not further augmented by depressive symptoms.

Mediation analysis showed that the strength of the association between depressive symptoms and mortality was not reduced by including inflammation, suggesting a direct effect of depressive symptoms on mortality risk (online Supplementary Table S1).

## Discussion

In this study, we examined the combined effect of depressive symptoms and inflammation on CVD and all-cause mortality in a large cohort of older adults. Our findings suggest that older men, with both depressive symptoms and high levels of inflammation, have an increased risk of CVD and all-cause mortality compared with men with depressive symptoms or inflammation alone. In addition, our study demonstrates the independent effects of depressive symptoms and inflammation on mortality, finding no evidence of either moderation or mediation. To our knowledge, our study is the first to investigate the combined effect of depressive symptoms and inflammation of mortality in both men and women.

### Depression, inflammation and mortality

We demonstrated an increased risk of all-cause mortality in the comparison between men with high and low baseline levels of inflammation. The addition of depression to the model increased the risk substantially suggestive of a particularly high-risk phenotype in men. This supports findings in healthy, older men showing that high inflammation predicted cardiovascular events only in people with depressed mood (Ladwig *et al*., 2005). These findings suggest that depression and inflammation might cause CVD through separate physiological pathways, such as elevated interleukin-6 upstream of CRP (Ridker, 2016), triglycerides (Parekh *et al*., 2017), cortisol as a result of stress-induced hyperactivity of the hypothalamic-pituitary-adrenal axis (Jokinen and Nordstrom, 2009), endothelial dysfunction (Chen *et al*., 2011) and platelet activation (Williams *et al*., 2014).

Our study found no significant interaction between depressive symptoms and inflammation on mortality and no effect of mediation, a finding which is supported by other studies but not by all. To our knowledge, there is only one other study which looked at the potential synergistic effect of depressive symptoms and inflammation in the prediction of cardiovascular events. Ladwig showed a significant interaction between depressive symptoms and inflammation in the prediction of cardiovascular events, suggesting a shared underlying mechanism (Ladwig *et al*., 2005). These authors, however, have only investigated men.

Most previous studies investigating the effects of inflammation mediating the association between depression and all-cause or CVD mortality showed either no or small effects. Similar to our study, Empana *et al*., (2005) showed that men with depressive symptoms had a 53% increase in the odds of CHD and the association remained unchanged when inflammatory markers were added to the model. Davidson *et al*., 2009 found that depressive symptoms increased the risk of incident CHD; this risk was not explained by increased inflammation in either men or women. Nabi *et al*., 2008 also found an association between psychological distress and incident CHD which remained after adjustment for inflammatory markers. Contrary to our study and the studies cited above, there has also been reports of mediation. Hughes *et al*., (2016) reported an association between depressive symptoms and all-cause mortality which was partly explained by inflammation (CRP 7.3%). Inflammation partly mediated the predictive value of depressive symptoms by 6.5% in cardiovascular mortality risk (Kop *et al*., 2010), and by 8.1% in cardiovascular hospitalization (Hiles *et al*., 2015). This supports the knowledge that other pathways are also involved in the depression and mortality link.

### Effect of gender on the link between depressive symptoms, inflammation and mortality

Clear sex-specific differences were observed in the inter-relationships of depressive symptoms, inflammation and mortality in this study. The combination of depressive symptoms and inflammation only conferred an increased mortality risk in men. A previous population cohort study investigating inflammation and mortality also showed sex differences. Ahmadi-Abhari *et al*., reported that high levels of CRP increased mortality risk in men at lower clinical threshold categories than in women, most notably for CVD mortality (2013). A similar trend has also been observed in the development of CHD. In a study of older men and women, Cushman *et al*. showed that the presence of high inflammation was predictive of CHD in men with intermediate Framingham Risk Scores, whereas it only became predictive of CHD in women with a high Framingham risk (2005). Furthermore, a large meta-analysis showed that inflammation only discriminated 10-year risk of cardiovascular events in men, but not in women (Kaptoge *et al*., 2012). Some reports on the association between depression and inflammation also support our findings, although not all. Two large population cohort studies have shown that major depression/depressive symptoms and inflammation are more strongly associated in men than in women (Ford and Erlinger, 2004; Elovainio *et al*., 2009). However in contrast, in a study of 508 healthy adults, depressive symptoms were only associated with inflammation in women, not in men (Ma *et al*., 2010). Furthermore, a study of women with suspected coronary ischemia demonstrated a robust association between depressive symptoms and inflammation which was not explained by CVD risk factors (Vaccarino *et al*., 2007).

Further speculation on gender differences is inspired by a recent review by Raison and Miller (2017). The authors propose that in evolutionary terms, depression may have provided an adaptive advantage to women. Inflammation was detrimental to fertility in ancestral environments (Van Bodegom *et al*., 2007; Schaller and Park, 2011; Kobayashi *et al*., 2013). Depressive symptoms promoted sickness behaviours (e.g. lethargy, psychomotor slowing and social withdrawal) which provided increased protection against pathogens (e.g. by conserving energy for an immune response), thereby reducing the need for a high inflammatory response. This is supported by findings which show that women demonstrate increased levels of depression in response to inflammatory challenge compared with men (Moieni *et al*., 2015; Udina *et al*., 2012). If women are more likely to develop depression in response to immune activation then it is possible that the presence of depressive symptoms is likely to reflect less severe underlying biological pathology compared with men and consequently lower mortality risk. Whilst this explanation is intuitively appealing, further research is required to confirm gender specific immune mechanisms in depression.

Another possible explanation for our observed sex differences is the influence of sex hormones, particularly the protective effect of oestrogen on the heart. In people under 75, the incident of cardiovascular-related death is lower in women than in men (British Heart Foundation, 2014), with the development of atherosclerosis occurring post menopause in 95% of women (Fairweather, 2014). Even at 65–74 years of age, almost two decades after the average occurrence of menopause, women have a substantially lower incidence of CVD compared with men. This suggests that former exposure to endogenous estrogen may be atheroprotective long after menopause. Women have also been shown to have longer telomeres than men (Gardner *et al*., 2014). Shorter telomeres have been associated with early death in the general population (Weischer *et al*., 2012) although findings are inconsistent (Bojesen, 2013). Telomere length has been more robustly associated with CHD, independently of traditional vascular risk factors (Haycock *et al*., 2014). It is not yet understood exactly why women differ from men in the development of CHD, however to date women have been underrepresented in cardiovascular trials, resulting in a bias towards factors which are relevant to disease aetiology in men (Fairweather, 2014).

Current National Institute for Health and Clinical Excellence (NICE) guidelines recommend the use of the QRISK2 risk assessment tool to assess risk for the primary prevention of CVD in both men and women up to 84 years (National Institute for Health and Care Excellence, 2014). There has been some debate as to whether or not the addition of circulating inflammatory markers, such as CRP, should now be included in screening measures for cardiovascular risk (Pearson *et al*., 2003; Peters *et al*., 2013). The main uncertainty seems to be whether the modest increases in risk associated with higher inflammation can produce significant health benefits. This is understandable when considering that population studies have shown that CRP is a relatively moderate predictor of CVD risk, yielding an odds ratio of 1.5 when comparing the top baseline tertile with the bottom (Danesh *et al*., 2004). In addition, when compared with traditional risk factors, such as smoking and total cholesterol, CRP only slightly improved their predictive value. Our study demonstrates that men with comorbid depressive symptoms and high inflammation constitute a clinically meaningful risk category. In light of these findings, it might be worth considering inflammation as a cardiovascular risk factor in depressed men. This could help identify patients who may benefit from targeted prophylactic intervention, improving screening efficacy and cardiovascular outcomes.

### Study strengths and limitations

The strengths of our study include the prospective design, the presence of both women and men and the nationally representative nature of the ELSA cohort. Despite this, our study also has limitations. The first limitation of this study is that we measured depressive symptoms and inflammation at only one-time point at wave 2 (2004–2005) and did not investigate any change in levels at a later point. This design was chosen in order to maximise the number of participants with inflammation and depressive symptoms at baseline and to allow a longer follow-up period to capture mortality.

Nevertheless, we did not find an association of depressive symptoms and mortality in the absence of inflammation. In a recent study using the same cohort, which considered depressive symptoms across several years, a dose-response association was observed between persistence of depressive symptoms across time and mortality risk (White *et al*., 2016). Similar to these findings, data from the Longitudinal Aging Study Amsterdam also found that transient depressive episodes did not predict mortality although chronic depression did (Geerlings *et al*., 2002). Secondly, we also did not control for medication use in our analysis, as this data was not available. Statins, for example, present anti-inflammatory effects (Antonopoulos *et al*., 2012) and therefore medication use may have interfered with current findings. Thirdly, our study had a smaller proportion of female deaths compared to male deaths and therefore it is unclear whether our lack of association in women reflects an absence of an effect or is a result of insufficient power. The only closest previous study to date investigated combined depressive symptoms and inflammation in the development of CVD and was restricted to men. Finally, in our study depressive symptoms were measured by an 8 item self-reported questionnaire, rather than a diagnostic interview. Short scales such as this have previously been criticised for a lack of specificity. To address this, we defined depressive symptoms using a conservative cut-off of 4, which increases the measure's ability to discriminate between true and false positives.

In conclusion, we demonstrated that men with concurrent depressive symptoms and increased inflammation constitute a high-mortality risk phenotype. This risk is particularly high for cardiovascular-related death. These findings have clinical implications for the treatment and prevention of depression and inflammation in men. Subgroups of depressed individuals with comorbid inflammation may benefit from additional anti-inflammatory pharmacotherapy. Further research is needed to investigate whether combined interventions improve outcomes.

## Supplementary material

For supplementary material accompanying this paper visit https://doi.org/10.1017/S003329171800209X. click here to view supplementary material

## References

[ref1] Ahmadi-Abhari S, Luben RN, Wareham NJ and Khaw KT (2013) Seventeen year risk of all-cause and cause-specific mortality associated with C-reactive protein, fibrinogen and leukocyte count in men and women: the EPIC-Norfolk study. European Journal of Epidemiology 28, 541–550. 23821244 10.1007/s10654-013-9819-6

[ref2] Anstey KJ and Luszcz MA (2002) Mortality risk varies according to gender and change in depressive status in very old adults. Psychosomatic Medicine 64, 880–888. 12461193 10.1097/01.psy.0000028827.64279.60

[ref3] Antonopoulos AS, Margaritis M, Lee R, Channon K and Antoniades C (2012) Statins as anti-inflammatory agents in atherogenesis: molecular mechanisms and lessons from the recent clinical trials. Current Pharmaceutical Design 18, 1519–1530. 22364136 10.2174/138161212799504803 PMC3394171

[ref4] Au B, Smith KJ, Gariepy G and Schmitz N (2015) The longitudinal associations between C-reactive protein and depressive symptoms: evidence from the English Longitudinal Study of Ageing (ELSA). International Journal of Geriatric Psychiatry 30, 976–984. 25537199 10.1002/gps.4250

[ref5] Banks J, Breeze E, Lessof C and Nazroo J (2006) Retirement, health and relationships of the older population in England: The 2004 English Longitudinal Study of Ageing (Wave 2).

[ref6] Barth J, Schumacher M and Herrmann-Lingen C (2004) Depression as a risk factor for mortality in patients with coronary heart disease: a meta-analysis. Psychosomatic Medicine 66, 802–813. 15564343 10.1097/01.psy.0000146332.53619.b2

[ref7] Bell JA, Kivimaki M, Bullmore ET, Steptoe A and Carvalho LA (2017) Repeated exposure to systemic inflammation and risk of new depressive symptoms among older adults. Translational Psychiatry 7, e1208. 28809860 10.1038/tp.2017.155 PMC5611724

[ref8] Biasucci LM, La Rosa G, Pedicino D, D'Aiello A, Galli M and Liuzzo G (2017) Where does inflammation fit? Current Cardiology Reports 19, 84. 28779286 10.1007/s11886-017-0896-0

[ref9] Bojesen SE (2013) Telomeres and human health. Journal of Internal Medicine 274, 399–413. 24127938 10.1111/joim.12083

[ref10] British Heart Foundation (2014) *Cardiovascular Disease Statistics* [Online]. Available: https://www.bhf.org.uk/research/heart-statistics/heart-statistics-publications/cardiovascular-disease-statistics-2015 [Accessed 8th Feb 2017].

[ref11] Callahan CM, Wolinsky FD, Stump TE, Nienaber NA, Hui SL and Tierney WM (1998) Mortality, symptoms, and functional impairment in late-life depression. Journal of General Internal Medicine 13, 746–752. 9824520 10.1046/j.1525-1497.1998.00226.x PMC1497028

[ref12] Chen H, Yiu KH and Tse HF (2011) Relationships between vascular dysfunction, circulating endothelial progenitor cells, and psychological status in healthy subjects. Depression and Anxiety 28, 719–727. 21681866 10.1002/da.20839

[ref13] Copeland WE, Shanahan L, Worthman C, Angold A and Costello EJ (2012) Cumulative depression episodes predict later C-reactive protein levels: a prospective analysis. Biological Psychiatry 71, 15–21. 22047718 10.1016/j.biopsych.2011.09.023 PMC3586231

[ref14] Correll CU, Solmi M, Veronese N, Bortolato B, Rosson S, Santonastaso P, Thapa-Chhetri N, Fornaro M, Gallicchio D, Collantoni E, Pigato G, Favaro A, Monaco F, Kohler C, Vancampfort D, Ward PB, Gaughran F, Carvalho AF and Stubbs B (2017) Prevalence, incidence and mortality from cardiovascular disease in patients with pooled and specific severe mental illness: a large-scale meta-analysis of 3 211 768 patients and 113 383 368 controls. World Psychiatry 16, 163–180. 28498599 10.1002/wps.20420 PMC5428179

[ref15] Cuijpers P (2001) Mortality and depressive symptoms in inhabitants of residential homes. International Journal of Geriatric Psychiatry 16, 131–138. 11241717 10.1002/1099-1166(200102)16:2<131::aid-gps283>3.0.co;2-w

[ref16] Cuijpers P and Smit F (2002) Excess mortality in depression: a meta-analysis of community studies. Journal of Affective Disorders 72, 227–236. 12450639 10.1016/s0165-0327(01)00413-x

[ref17] Cushman M, Arnold AM, Psaty BM, Manolio TA, Kuller LH, Burke GL, Polak JF and Tracy RP (2005) C-reactive protein and the 10-year incidence of coronary heart disease in older men and women: the cardiovascular health study. Circulation 112, 25–31. 15983251 10.1161/CIRCULATIONAHA.104.504159

[ref18] Danesh J, Wheeler JG, Hirschfield GM, Eda S, Eiriksdottir G, Rumley A, Lowe GD, Pepys MB and Gudnason V (2004) C-reactive protein and other circulating markers of inflammation in the prediction of coronary heart disease. The New England Journal of Medicine 350, 1387–1397. 15070788 10.1056/NEJMoa032804

[ref19] Davidson KW, Schwartz JE, Kirkland SA, Mostofsky E, Fink D, Guernsey D and Shimbo D (2009) Relation of inflammation to depression and incident coronary heart disease (from the Canadian Nova Scotia Health Survey [NSHS95] Prospective Population Study). The American Journal of Cardiology 103, 755–761. 19268727 10.1016/j.amjcard.2008.11.035 PMC2905847

[ref20] Demakakos P, Cooper R, Hamer M, de Oliveira C, Hardy R and Breeze E (2013) The bidirectional association between depressive symptoms and gait speed: evidence from the English Longitudinal Study of Ageing (ELSA). PLoS ONE 8, e68632. 23874698 10.1371/journal.pone.0068632 PMC3706406

[ref21] Diniz BS, Reynolds CF IIIrd, Butters MA, Dew MA, Firmo JO, Lima-Costa MF and Castro-Costa E (2014) The effect of gender, age, and symptom severity in late-life depression on the risk of all-cause mortality: the Bambui Cohort Study of Aging. Depression and Anxiety 31, 787–795. 24353128 10.1002/da.22226 PMC4062606

[ref22] Dowlati Y, Herrmann N, Swardfager W, Liu H, Sham L, Reim EK and Lanctot KL (2010) A meta-analysis of cytokines in major depression. Biological Psychiatry 67, 446–457. 20015486 10.1016/j.biopsych.2009.09.033

[ref23] Elovainio M, Aalto AM, Kivimaki M, Pirkola S, Sundvall J, Lonnqvist J and Reunanen A (2009) Depression and C-reactive protein: population-based Health 2000 Study. Psychosomatic Medicine 71, 423–430. 19297307 10.1097/PSY.0b013e31819e333a

[ref24] Empana JP, Sykes DH, Luc G, Juhan-Vague I, Arveiler D, Ferrieres J, Amouyel P, Bingham A, Montaye M, Ruidavets JB, Haas B, Evans A, Jouven X and Ducimetiere P (2005) Contributions of depressive mood and circulating inflammatory markers to coronary heart disease in healthy European men: the Prospective Epidemiological Study of Myocardial Infarction (PRIME). Circulation 111, 2299–2305. 15867179 10.1161/01.CIR.0000164203.54111.AE

[ref25] Eyre HA, Air T, Pradhan A, Johnston J, Lavretsky H, Stuart MJ and Baune BT (2016) A meta-analysis of chemokines in major depression. Progress in Neuro-Psychopharmacology & Biological Psychiatry 68, 1–8. 26903140 10.1016/j.pnpbp.2016.02.006 PMC5536843

[ref26] Fairweather D (2014) Sex differences in inflammation during atherosclerosis. Clinical Medicine Insights. Cardiology 8, 49–59. 10.4137/CMC.S17068 PMC4405090 25983559

[ref27] Ford DE and Erlinger TP (2004) Depression and C-reactive protein in US adults: data from the Third National Health and Nutrition Examination Survey. Archives of Internal Medicine 164, 1010–1014. 15136311 10.1001/archinte.164.9.1010

[ref28] Gan Y, Gong Y, Tong X, Sun H, Cong Y, Dong X, Wang Y, Xu X, Yin X, Deng J, Li L, Cao S and Lu Z (2014) Depression and the risk of coronary heart disease: a meta-analysis of prospective cohort studies. BMC Psychiatry 14, 371. 25540022 10.1186/s12888-014-0371-z PMC4336481

[ref29] Gardner M, Bann D, Wiley L, Cooper R, Hardy R, Nitsch D, Martin-Ruiz C, Shiels P, Sayer AA, Barbieri M, Bekaert S, Bischoff C, Brooks-Wilson A, Chen W, Cooper C, Christensen K, De Meyer T, Deary I, Der G, Diez Roux A, Fitzpatrick A, Hajat A, Halaschek-Wiener J, Harris S, Hunt SC, Jagger C, Jeon HS, Kaplan R, Kimura M, Lansdorp P, Li C, Maeda T, Mangino M, Nawrot TS, Nilsson P, Nordfjall K, Paolisso G, Ren F, Riabowol K, Robertson T, Roos G, Staessen JA, Spector T, Tang N, Unryn B, van der Harst P, Woo J, Xing C, Yadegarfar ME, Park JY, Young N, Kuh D, von Zglinicki T and Ben-Shlomo Y (2014) Gender and telomere length: systematic review and meta-analysis. Experimental Gerontology 51, 15–27. 24365661 10.1016/j.exger.2013.12.004 PMC4523138

[ref30] Geerlings SW, Beekman AT, Deeg DJ, Twisk JW and Van Tilburg W (2002) Duration and severity of depression predict mortality in older adults in the community. Psychological Medicine 32, 609–618. 12102375 10.1017/s0033291702005585

[ref31] Gimeno D, Kivimaki M, Brunner EJ, Elovainio M, De Vogli R, Steptoe A, Kumari M, Lowe GD, Rumley A, Marmot MG and Ferrie JE (2009) Associations of C-reactive protein and interleukin-6 with cognitive symptoms of depression: 12-year follow-up of the Whitehall II study. Psychological Medicine 39, 413–423. 18533059 10.1017/S0033291708003723 PMC2788760

[ref32] Golia E, Limongelli G, Natale F, Fimiani F, Maddaloni V, Pariggiano I, Bianchi R, Crisci M, D'Acierno L, Giordano R, Di Palma G, Conte M, Golino P, Russo MG, Calabro R and Calabro P (2014) Inflammation and cardiovascular disease: from pathogenesis to therapeutic target. Current Atherosclerosis Reports 16, 435. 25037581 10.1007/s11883-014-0435-z

[ref33] Haapakoski R, Mathieu J, Ebmeier KP, Alenius H and Kivimaki M (2015) Cumulative meta-analysis of interleukins 6 and 1beta, tumour necrosis factor alpha and C-reactive protein in patients with major depressive disorder. Brain, Behavior, & Immunity 49, 206–215. 10.1016/j.bbi.2015.06.001 PMC4566946 26065825

[ref34] Hamer M, Bates CJ and Mishra GD (2011) Depression, physical function, and risk of mortality: National Diet and Nutrition Survey in adults older than 65 years. American Journal of Geriatric Psychiatry 19, 72–78. 20808095 10.1097/JGP.0b013e3181df465e

[ref35] Hamer M, Molloy GJ, de Oliveira C and Demakakos P (2009) Persistent depressive symptomatology and inflammation: to what extent do health behaviours and weight control mediate this relationship? Brain, Behavior, & Immunity 23, 413–418. 10.1016/j.bbi.2009.01.005 19486658

[ref36] Hamer M, Batty GD and Kivimaki M (2012) Risk of future depression in people who are obese but metabolically healthy: The English Longitudinal Study of Ageing. Molecular Psychiatry 17, 940–945. 22525487 10.1038/mp.2012.30 PMC3428506

[ref37] Haycock PC, Heydon EE, Kaptoge S, Butterworth AS, Thompson A and Willeit P (2014) Leucocyte telomere length and risk of cardiovascular disease: systematic review and meta-analysis. British Medical Journal *(*Clinical Research Edition*)* 349, g4227. 10.1136/bmj.g4227 PMC4086028 25006006

[ref38] Hiles SA, Baker AL, de Malmanche T, McEvoy M, Boyle M and Attia J (2015) The role of inflammatory markers in explaining the association between depression and cardiovascular hospitalisations. Journal of Behavioral Medicine 38, 609–619. 25835436 10.1007/s10865-015-9637-2

[ref39] Howren MB, Lamkin DM and Suls J (2009) Associations of depression with C-reactive protein, IL-1, and IL-6: a meta-analysis. Psychosomatic Medicine 71, 171–186. 19188531 10.1097/PSY.0b013e3181907c1b

[ref40] Hughes MF, Patterson CC, Appleton KM, Blankenberg S, Woodside JV, Donnelly M, Linden G, Zeller T, Esquirol Y and Kee F (2016) The predictive value of depressive symptoms for all-cause mortality: findings from the PRIME Belfast study examining the role of inflammation and cardiovascular risk markers. Psychosomatic Medicine 78, 401–411. 26761713 10.1097/PSY.0000000000000289

[ref41] Hybels CF, Pieper CF and Blazer DG (2002) Sex differences in the relationship between subthreshold depression and mortality in a community sample of older adults. The American Journal of Geriatric Psychiatry 10, 283–291. 11994215

[ref42] Irwin MR and Miller AH (2007) Depressive disorders and immunity: 20 years of progress and discovery. Brain, Behavior, & Immunity 21, 374–383. 10.1016/j.bbi.2007.01.010 17360153

[ref43] Jeong HG, Lee JJ, Lee SB, Park JH, Huh Y, Han JW, Kim TH, Chin HJ and Kim KW (2013) Role of severity and gender in the association between late-life depression and all-cause mortality. International Psychogeriatrics 25, 677–684. 23256908 10.1017/S1041610212002190

[ref44] Jokinen J and Nordstrom P (2009) HPA axis hyperactivity and cardiovascular mortality in mood disorder inpatients. Journal of Affective Disorders 116, 88–92. 19054568 10.1016/j.jad.2008.10.025

[ref45] Joynt KE, Whellan DJ and O'Connor CM (2003) Depression and cardiovascular disease: mechanisms of interaction. Biological Psychiatry 54, 248–261. 12893101 10.1016/s0006-3223(03)00568-7

[ref46] Kaptoge S, Di Angelantonio E, Pennells L, Wood AM, White IR, Gao P, Walker M, Thompson A, Sarwar N, Caslake M, Butterworth AS, Amouyel P, Assmann G, Bakker SJ, Barr EL, Barrett-Connor E, Benjamin EJ, Bjorkelund C, Brenner H, Brunner E, Clarke R, Cooper JA, Cremer P, Cushman M, Dagenais GR, D'Agostino RB Sr., Dankner R, Davey-Smith G, Deeg D, Dekker JM, Engstrom G, Folsom AR, Fowkes FG, Gallacher J, Gaziano JM, Giampaoli S, Gillum RF, Hofman A, Howard BV, Ingelsson E, Iso H, Jorgensen T, Kiechl S, Kitamura A, Kiyohara Y, Koenig W, Kromhout D, Kuller LH, Lawlor DA, Meade TW, Nissinen A, Nordestgaard BG, Onat A, Panagiotakos DB, Psaty BM, Rodriguez B, Rosengren A, Salomaa V, Kauhanen J, Salonen JT, Shaffer JA, Shea S, Ford I, Stehouwer CD, Strandberg TE, Tipping RW, Tosetto A, Wassertheil-Smoller S, Wennberg P, Westendorp RG, Whincup PH, Wilhelmsen L, Woodward M, Lowe GD, Wareham NJ, Khaw KT, Sattar N, Packard CJ, Gudnason V, Ridker PM, Pepys MB, Thompson SG and Danesh J (2012) C-reactive protein, fibrinogen, and cardiovascular disease prediction. The New England Journal of Medicine 367, 1310–1320. 23034020 10.1056/NEJMoa1107477 PMC3714101

[ref47] Kobayashi K, Uejyo T, Oyama S, Rahman MM and Kumura H (2013) Histological analysis of mammary gland remodeling caused by lipopolysaccharide in lactating mice. Cell and Tissue Research 354, 495–506. 23881408 10.1007/s00441-013-1688-5

[ref48] Kop WJ, Stein PK, Tracy RP, Barzilay JI, Schulz R and Gottdiener JS (2010) Autonomic nervous system dysfunction and inflammation contribute to the increased cardiovascular mortality risk associated with depression. Psychosomatic Medicine 72, 626–635. 20639389 10.1097/PSY.0b013e3181eadd2b PMC3059072

[ref49] Ladwig KH, Marten-Mittag B, Lowel H, Doring A and Koenig W (2005) C-reactive protein, depressed mood, and the prediction of coronary heart disease in initially healthy men: results from the MONICA-KORA Augsburg Cohort Study 1984–1998. European Heart Journal 26, 2537–2542. 16126719 10.1093/eurheartj/ehi456

[ref50] Lasserre AM, Marti-Soler H, Strippoli MP, Vaucher J, Glaus J, Vandeleur CL, Castelao E, Marques-Vidal P, Waeber G, Vollenweider P and Preisig M (2016) Clinical and course characteristics of depression and all-cause mortality: a prospective population-based study. Journal of Affective Disorders 189, 17–24. 26402343 10.1016/j.jad.2015.09.010

[ref51] Laursen TM, Munk-Olsen T, Nordentoft M and Mortensen PB (2007) Increased mortality among patients admitted with major psychiatric disorders: a register-based study comparing mortality in unipolar depressive disorder, bipolar affective disorder, schizoaffective disorder, and schizophrenia. The Journal of Clinical Psychiatry 68, 899–907. 17592915 10.4088/jcp.v68n0612

[ref52] Liu Y, Ho RC and Mak A (2012) Interleukin (IL)-6, tumour necrosis factor alpha (TNF-alpha) and soluble interleukin-2 receptors (sIL-2R) are elevated in patients with major depressive disorder: a meta-analysis and meta-regression. Journal of Affective Disorders 139, 230–239. 21872339 10.1016/j.jad.2011.08.003

[ref53] Ma Y, Chiriboga DE, Pagoto SL, Rosal MC, Li W, Merriam PA, Hebert JR, Whited MC and Ockene IS (2010) Association between depression and C-reactive protein. Cardiology Research & Practice 2011, 286509. 21234098 10.4061/2011/286509 PMC3014664

[ref54] Maes M, Berk M, Goehler L, Song C, Anderson G, Gałecki P and Leonard B (2012) Depression and sickness behavior are Janus-faced responses to shared inflammatory pathways. BMC Medicine 10, 66. 22747645 10.1186/1741-7015-10-66 PMC3391987

[ref55] Malgaroli M, Galatzer-Levy IR and Bonanno GA (2017) Heterogeneity in trajectories of depression in response to divorce is associated with differential risk for mortality. Clinical Psychological Science 5, 843–850. 29034135 10.1177/2167702617705951 PMC5637453

[ref56] Marmot M, Allen J, Bell R, Bloomer E and Goldblatt P (2012) WHO European review of social determinants of health and the health divide. Lancet 380, 1011–1029. 22964159 10.1016/S0140-6736(12)61228-8

[ref57] McCusker J, Cole M, Ciampi A, Latimer E, Windholz S and Belzile E (2006) Does depression in older medical inpatients predict mortality? Journals of Gerontology Series A-Biological Sciences & Medical Sciences 61, 975–981. 10.1093/gerona/61.9.975 16960030

[ref58] Meijer A, Conradi HJ, Bos EH, Thombs BD, van Melle JP and de Jonge P (2011) Prognostic association of depression following myocardial infarction with mortality and cardiovascular events: a meta-analysis of 25 years of research. General Hospital Psychiatry 33, 203–216. 21601716 10.1016/j.genhosppsych.2011.02.007

[ref59] Meijer A, Conradi HJ, Bos EH, Anselmino M, Carney RM, Denollet J, Doyle F, Freedland KE, Grace SL, Hosseini SH, Lane DA, Pilote L, Parakh K, Rafanelli C, Sato H, Steeds RP, Welin C and de Jonge P (2013) Adjusted prognostic association of depression following myocardial infarction with mortality and cardiovascular events: individual patient data meta-analysis. British Journal of Psychiatry 203, 90–102. 23908341 10.1192/bjp.bp.112.111195

[ref60] Mhaoláin AMN, Fan CW, Romero-Ortuno R, Cogan L, Cunningham C, Kenny R-A and Lawlor B (2012) Frailty, depression, and anxiety in later life. International Psychogeriatrics 24, 1265–1274. 22333477 10.1017/S1041610211002110

[ref61] Miller AH and Raison CL (2016) The role of inflammation in depression: from evolutionary imperative to modern treatment target. Nature reviews. Immunology 16, 22–34. 10.1038/nri.2015.5 PMC5542678 26711676

[ref62] Miller AH, Maletic V and Raison CL (2009) Inflammation and Its discontents: the role of cytokines in the pathophysiology of major depression. Biological Psychiatry 65, 732–741. 19150053 10.1016/j.biopsych.2008.11.029 PMC2680424

[ref63] Missinne S, Vandeviver C, Van de Velde S and Bracke P (2014) Measurement equivalence of the CES-D 8 depression-scale among the ageing population in eleven European countries. Social Science Research 46, 38–47. 24767588 10.1016/j.ssresearch.2014.02.006

[ref64] Moieni M, Irwin MR, Jevtic I, Olmstead R, Breen EC and Eisenberger NI (2015) Sex differences in depressive and socioemotional responses to an inflammatory challenge: implications for sex differences in depression. Neuropsychopharmacology 40, 1709. 25598426 10.1038/npp.2015.17 PMC4915253

[ref65] Nabi H, Singh-Manoux A, Shipley M, Gimeno D, Marmot MG and Kivimaki M (2008) Do psychological factors affect inflammation and incident coronary heart disease: the Whitehall II Study. Arteriosclerosis, Thrombosis, and Vascular Biology 28, 1398–1406. 10.1161/ATVBAHA.108.167239 PMC12825741 18436803

[ref66] National Institute for Health and Care Excellence (2014) *Cardiovascular disease: risk assessment and reduction, including lipid modification* [Online]. Available: https://www.nice.org.uk/guidance/cg181/chapter/1-Recommendations#identifying-and-assessing-cardiovascular-disease-cvd-risk-2 [Accessed 14/3/2016 2016].

[ref67] Nicholson A, Kuper H and Hemingway H (2006) Depression as an aetiologic and prognostic factor in coronary heart disease: a meta-analysis of 6362 events among 146 538 participants in 54 observational studies. European Heart Journal 27, 2763–2774. 17082208 10.1093/eurheartj/ehl338

[ref68] Parekh A, Smeeth D, Milner Y and Thure S (2017) The role of lipid biomarkers in major depression. Healthcare *(*Basel*)* 5, 5. 10.3390/healthcare5010005 PMC5371911 28165367

[ref69] Pearson TA, Mensah GA, Alexander RW, Anderson JL, Cannon RO, Criqui M, Fadl YY, Fortmann SP, Hong Y and Myers GL (2003) Markers of inflammation and cardiovascular disease application to clinical and public health practice: a statement for healthcare professionals from the centers for disease control and prevention and the American Heart Association. Circulation 107, 499–511. 12551878 10.1161/01.cir.0000052939.59093.45

[ref70] Penninx BW, Guralnik JM, Mendes de Leon CF, Pahor M, Visser M, Corti MC and Wallace RB (1998) Cardiovascular events and mortality in newly and chronically depressed persons >70 years of age. The American Journal of Cardiology 81, 988–994. 9576158 10.1016/s0002-9149(98)00077-0

[ref71] Peters SAE, Visseren FLJ and Grobbee DE (2013) Biomarkers: screening for C-reactive protein in CVD prediction. Nature Reviews Cardiology 10, 12–14. 10.1038/nrcardio.2012.164 23165068

[ref72] Piccinelli M and Wilkinson G (2000) Gender differences in depression. Critical review. British Journal of Psychiatry 177, 486–492. 11102321 10.1192/bjp.177.6.486

[ref73] Pletcher MJ and Moran AE (2017) Cardiovascular risk assessment. Medical Clinics of North America 101, 673–688. 28577619 10.1016/j.mcna.2017.03.002

[ref74] Radloff LS (1977) The CES-D scale a self-report depression scale for research in the general population. Applied Psychological Measurement 1, 385–401.

[ref75] Raison CL and Miller AH (2017) Pathogen–host defense in the evolution of depression: insights into epidemiology, genetics, bioregional differences and female preponderance. Neuropsychopharmacology 42, 5–27. 27629366 10.1038/npp.2016.194 PMC5143499

[ref76] Raison CL, Capuron L and Miller AH (2006) Cytokines sing the blues: inflammation and the pathogenesis of depression. Trends in Immunology 27, 24–31. 16316783 10.1016/j.it.2005.11.006 PMC3392963

[ref78] Ridker P (2016) From C-reactive protein to interleukin-6 to interleukin-1: moving upstream to identify novel targets for atheroprotection. Circulation Research 118, 145–156. 26837745 10.1161/CIRCRESAHA.115.306656 PMC4793711

[ref79] Rugulies R (2002) Depression as a predictor for coronary heart disease: a review and meta-analysis1 1The full text of this article is available via AJPM Online at www. Ajpm-online. net. American Journal of Preventive Medicine 23, 51–61. 12093424 10.1016/s0749-3797(02)00439-7

[ref80] Russ TC, Stamatakis E, Hamer M, Starr JM, Kivimaki M and Batty GD (2012) Association between psychological distress and mortality: individual participant pooled analysis of 10 prospective cohort studies. British Medical Journal *(*Clinical Research Edition*)* 345, e4933. 10.1136/bmj.e4933 PMC3409083 22849956

[ref81] Ryan J, Carriere I, Ritchie K, Stewart R, Toulemonde G, Dartigues JF, Tzourio C and Ancelin ML (2008) Late-life depression and mortality: influence of gender and antidepressant use. British Journal of Psychiatry 192, 12–18. 18174502 10.1192/bjp.bp.107.039164

[ref82] Schaller M and Park JH (2011) The behavioral immune system (and why it matters). Current Directions in Psychological Science 20, 99–103.

[ref83] Schoevers RA, Geerlings MI, Deeg DJ, Holwerda TJ, Jonker C and Beekman AT (2009) Depression and excess mortality: evidence for a dose response relation in community living elderly. International Journal of Geriatric Psychiatry 24, 169–176. 18642389 10.1002/gps.2088

[ref84] Schulz R, Drayer RA and Rollman BL (2002) Depression as a risk factor for non-suicide mortality in the elderly. Biological Psychiatry 52, 205–225. 12182927 10.1016/s0006-3223(02)01423-3

[ref85] Shimbo D, Chaplin W, Crossman D, Haas D and Davidson KW (2005) Role of depression and inflammation in incident coronary heart disease events. The American Journal of Cardiology 96, 1016–1021. 16188535 10.1016/j.amjcard.2005.05.064

[ref86] Steffick DE (2000) Documentation of Affective Functioning Measures in the Health and Retirement Study [Online]. Ann Arbor, MI: Survey Research Center, University of Michigan Available: http://hrsonline.isr.umich.edu/sitedocs/userg/dr-005.pdf [Accessed July 2017].

[ref87] Surtees PG, Wainwright NW, Boekholdt SM, Luben RN, Wareham NJ and Khaw KT (2008) Major depression, C-reactive protein, and incident ischemic heart disease in healthy men and women. Psychosomatic Medicine 70, 850–855. 18725428 10.1097/PSY.0b013e318183acd5

[ref88] Teng PR, Yeh CJ, Lee MC, Lin HS and Lai TJ (2013) Change in depressive status and mortality in elderly persons: results of a national longitudinal study. Archives of Gerontology & Geriatrics 56, 244–249. 22974662 10.1016/j.archger.2012.08.006

[ref89] Thombs BD, Bass EB, Ford DE, Stewart KJ, Tsilidis KK, Patel U, Fauerbach JA, Bush DE and Ziegelstein RC (2006) Prevalence of depression in survivors of acute myocardial infarction. Journal of General Internal Medicine 21, 30–38. 16423120 10.1111/j.1525-1497.2005.00269.x PMC1484630

[ref90] Udina M, Castellví P, Moreno-España J, Navinés R, Valdés M, Forns X, Langohr K, Sola R, Vieta E and Martín-Santos R (2012) Interferon-induced depression in chronic hepatitis C: a systematic review and meta-analysis. 10.4088/JCP.12r07694 22967776

[ref91] Vaccarino V, Johnson BD, Sheps DS, Reis SE, Kelsey SF, Bittner V, Rutledge T, Shaw LJ, Sopko G and Bairey Merz CN (2007) Depression, inflammation, and incident cardiovascular disease in women with suspected coronary ischemia: the National Heart, Lung, and Blood Institute-sponsored WISE study. Journal of the American College of Cardiology 50, 2044–2050. 18021871 10.1016/j.jacc.2007.07.069

[ref92] Valkanova V, Ebmeier KP and Allan CL (2013) CRP, IL-6 and depression: a systematic review and meta-analysis of longitudinal studies. Journal of Affective Disorders 150, 736–744. 23870425 10.1016/j.jad.2013.06.004

[ref93] Van Bodegom D, May L, Meij J and Westendorp RG (2007) Regulation of human life histories. Annals of the New York Academy of Sciences 1100, 84–97. 17460167 10.1196/annals.1395.007

[ref94] Van de Velde S, Levecque K and Bracke P (2009) Measurement equivalence of the CES-D 8 in the general population in Belgium: a gender perspective. Archives of Public Health 67, 15.

[ref95] Van der Kooy K, van Hout H, Marwijk H, Marten H, Stehouwer C and Beekman A (2007) Depression and the risk for cardiovascular diseases: systematic review and meta analysis. International Journal of Geriatric Psychiatry 22, 613–626. 17236251 10.1002/gps.1723

[ref96] van Melle JP, de Jonge P, Spijkerman TA, Tijssen JG, Ormel J, van Veldhuisen DJ, van den Brink RH and van den Berg MP (2004) Prognostic association of depression following myocardial infarction with mortality and cardiovascular events: a meta-analysis. Psychosomatic Medicine 66, 814–822. 15564344 10.1097/01.psy.0000146294.82810.9c

[ref97] Vinkers DJ, Stek ML, Gussekloo J, Van Der Mast RC and Westendorp RG (2004) Does depression in old age increase only cardiovascular mortality? The Leiden 85-plus Study. International Journal of Geriatric Psychiatry 19, 852–857. 15352142 10.1002/gps.1169

[ref98] Weischer M, Bojesen SE, Cawthon RM, Freiberg JJ, Tybjaerg-Hansen A and Nordestgaard BG (2012) Short telomere length, myocardial infarction, ischemic heart disease, and early death. Arteriosclerosis, Thrombosis, and Vascular Biology 32, 822–829. 10.1161/ATVBAHA.111.237271 22199369

[ref99] White J, Zaninotto P, Walters K, Kivimaki M, Demakakos P, Shankar A, Kumari M, Gallacher J and Batty GD (2015) Severity of depressive symptoms as a predictor of mortality: the English longitudinal study of ageing. Psychological Medicine 45, 2771–2779. 25936473 10.1017/S0033291715000732

[ref100] White J, Zaninotto P, Walters K, Kivimaki M, Demakakos P, Biddulph J, Kumari M, De Oliveira C, Gallacher J and Batty GD (2016) Duration of depressive symptoms and mortality risk: the English Longitudinal Study of Ageing (ELSA). British Journal of Psychiatry 208, 337–342. 26795425 10.1192/bjp.bp.114.155333 PMC4816969

[ref101] Williams MS, Rogers HL, Wang NY and Ziegelstein RC (2014) Do platelet-derived microparticles play a role in depression, inflammation, and acute coronary syndrome? Psychosomatics 55, 252–260. 24374086 10.1016/j.psym.2013.09.004 PMC4004656

[ref103] Wong ND (2014) Epidemiological studies of CHD and the evolution of preventive cardiology. Nature Reviews Cardiology 11, 276–289. 24663092 10.1038/nrcardio.2014.26

[ref104] Wu Q and Kling JM (2016) Depression and the risk of myocardial infarction and coronary death: a meta-analysis of prospective cohort studies. Medicine 95, e2815. 26871852 10.1097/MD.0000000000002815 PMC4753948

[ref105] Wulsin LR and Singal BM (2003) Do depressive symptoms increase the risk for the onset of coronary disease? A systematic quantitative review. Psychosomatic Medicine 65, 201–210. 12651987 10.1097/01.psy.0000058371.50240.e3

[ref106] Wulsin LR, Vaillant GE and Wells VE (1999) A systematic review of the mortality of depression. Psychosomatic Medicine 61, 6–17. 10024062 10.1097/00006842-199901000-00003

[ref107] Wulsin LR, Evans JC, Vasan RS, Murabito JM, Kelly-Hayes M and Benjamin EJ (2005) Depressive symptoms, coronary heart disease, and overall mortality in the Framingham Heart Study. Psychosomatic Medicine 67, 697–702. 16204426 10.1097/01.psy.0000181274.56785.28

[ref108] Zalli A, Jovanova O, Hoogendijk WJ, Tiemeier H and Carvalho LA (2015) Low-grade inflammation predicts persistence of depressive symptoms. Psychopharmacology 233, 1669–1678. 25877654 10.1007/s00213-015-3919-9 PMC4828485

