# Supplementary material for: Combined influence of depressive symptoms and systemic inflammation on all-cause and cardiovascular mortality: evidence for differential effects by gender in the English Longitudinal Study of Ageing
Source: Psychol Med. 2018 Sep 17;49(9):1521–31. doi: 10.1017/S003329171800209X (PMC6541870; doi:10.1017/S003329171800209X)
Supplement: Supplementary file 1 [file S003329171800209Xsup.zip › S003329171800209Xsup003.docx]

Supplementary table 1: Mediation analysis (n=5,328)

| **Mediation analyses** | | | | | | | |
| --- | --- | --- | --- | --- | --- | --- | --- |
|  | **Model 1^a^** | | **Model 1 + dichotomous CRP^b^** | | **Model 1 + continuous CRP^c^** | |  |
| **Men** | | | | | | |  |
| **All-cause mortality (420 deaths)** | | | | | | | |
| HR (95% CI) | 1.61 (1.06-2.44) | | | 1.56 (1.03-2.37) | | 1.65 (1.08-2.50) | |
| **Cardiovascular mortality (112 deaths)** | | | | | | | |
| HR (95% CI) | | 1.73 (0.83-3.61) | | 1.59 (0.76-3.29) | | 1.82 (0.73-3.79) | |
| **Women** |  | | |  | |  | |
| **All-cause mortality (334 deaths)** |  | | |  | |  | |
| HR (95% CI) | 1.01 (0.70-1.45) | | | 1.02 (0.71-1.46) | | 1.00 (0.69-1.44) | |
| **Cardiovascular mortality (109 deaths)** |  | | |  | |  | |
| HR (95% CI) | 0.55 (0.27-1.13) | | | 0.55 (0.27-1.14) | | 0.55 (0.27-1.13) | |

HR = Hazard ratio; CI = Confidence interval; CRP= C-reactive protein

Mediation analysis models, stratified by sex, are adjusted as follows: ^a^ Chronic symptoms of depression (wave 1 and wave 2) as main effects, plus adjustment for age, socioeconomic variables (marital status, level of education, household wealth) and chronic disease (cardiovascular disease, cancers, chronic lung disease); ^b^ as model, 1 plus adjustment for CRP dichotomised into two categories: <3mg/L defined as normal and 3-20mg/L defined as high; and ^c^ as model 1, plus adjustment for continuous CRP.
