# Supplementary material for: Combined influence of depressive symptoms and systemic inflammation on all-cause and cardiovascular mortality: evidence for differential effects by gender in the English Longitudinal Study of Ageing
Source: Psychol Med. 2018 Sep 17;49(9):1521–31. doi: 10.1017/S003329171800209X (PMC6541870; doi:10.1017/S003329171800209X)
Supplement: Supplementary file 1 [file S003329171800209Xsup.zip › S003329171800209Xsup002.docx]

**Appendix 1 Center for Epidemiologic Studies Depression Scale (CES-D 8)**

Study participants were asked about the occurrence (yes/no) of eight depressive symptoms:

“Now think about the past week and the feelings you have experienced. Please tell me if each of the following was true for you much of the time during the past week”:

1. Much of the time during the past week, you felt depressed?

1 Yes

2 No

1. Much of the time during the past week, you felt that everything you did was an effort?

1 Yes

2 No

1. Much of the time during the past week), your sleep was restless?

1 Yes

2 No

1. Much of the time during the past week, you were happy?

1 Yes

2 No

1. Much of the time during the past week, you felt lonely?

1 Yes
2 No

1. Much of the time during the past week, you enjoyed life?

1 Yes
2 No

1. Much of the time during the past week, you felt sad?

1 Yes
2 No

1. (Much of the time during the past week), you could not get going?

1 Yes
2 No

Each negative item is given a score of 1 (positive variables d and f are reverse scored) to give a total score ranging between 0 (no symptoms) and 8 (all eight symptoms).
