# Supplementary material for: Combined influence of depressive symptoms and systemic inflammation on all-cause and cardiovascular mortality: evidence for differential effects by gender in the English Longitudinal Study of Ageing
Source: Psychol Med. 2018 Sep 17;49(9):1521–31. doi: 10.1017/S003329171800209X (PMC6541870; doi:10.1017/S003329171800209X)
Supplement: Supplementary file 1 [file S003329171800209Xsup.zip › S003329171800209Xsup001.docx]

Supplementary figure 1: Kaplan-Meier Survival curves for all-cause and CVD mortality, stratified by sex.

(A) (B)


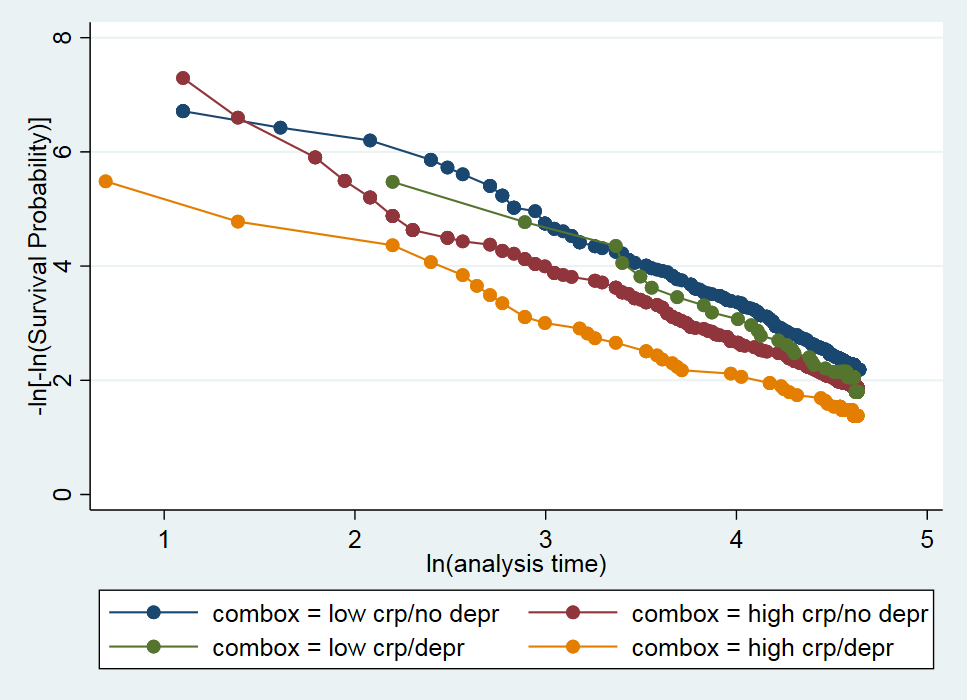

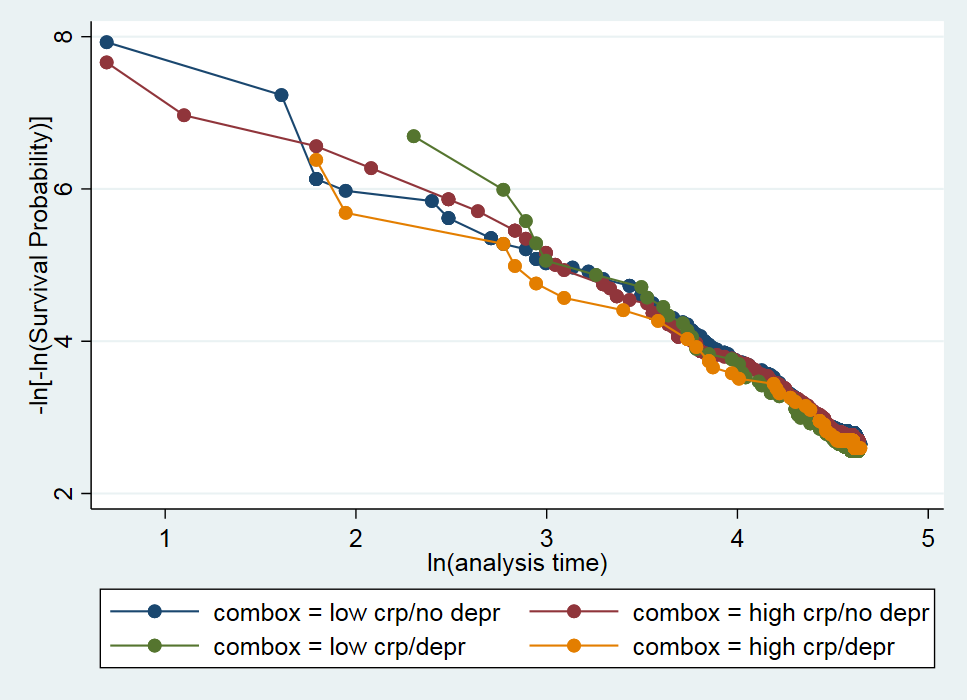


(C) (D)


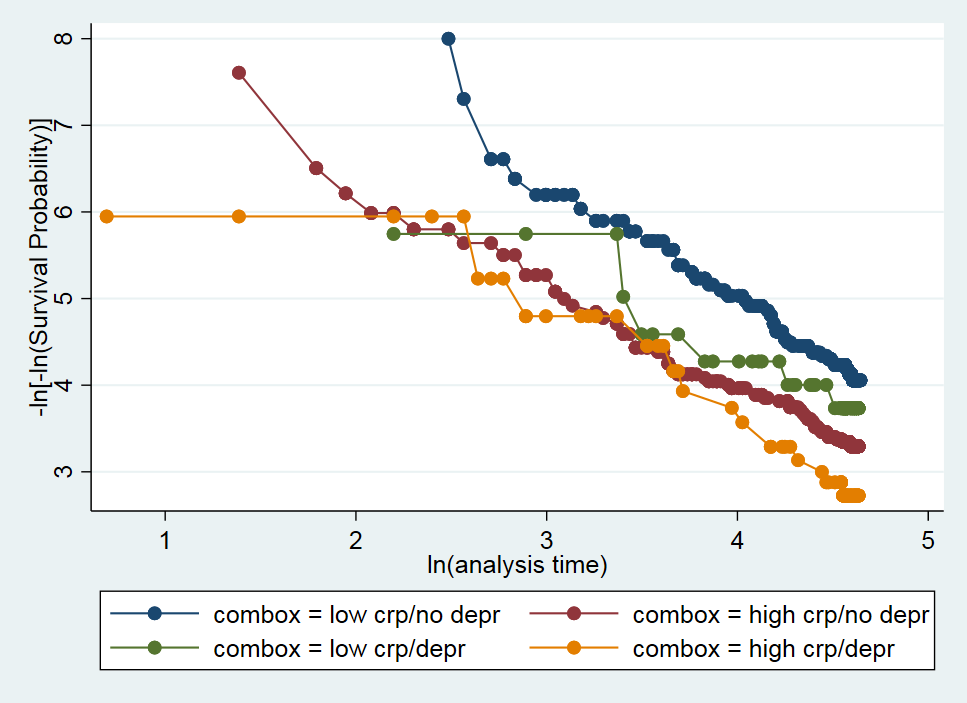

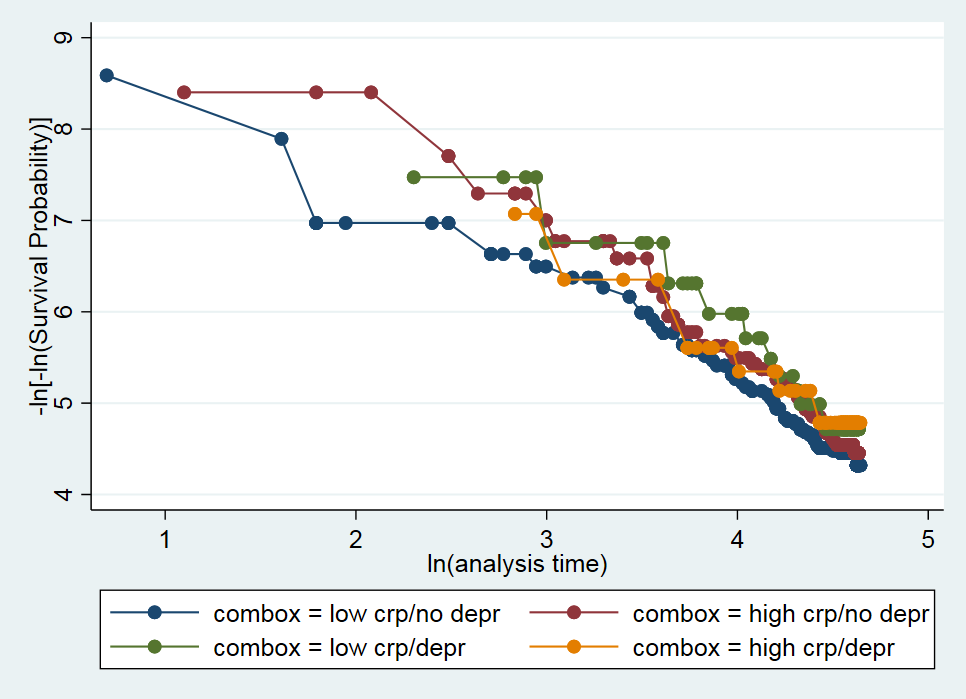


CRP = C-reactive protein; depr = depressive symptoms; CVD = cardiovascular disease. (A) = all-cause mortality in men; (B) = all-cause mortality in women; (C) = CVD mortality in men; (D) = CVD mortality in women.
